# Assessing awareness of long-term health risks among women with a history of preeclampsia: a cross-sectional study

**DOI:** 10.3389/fmed.2023.1236314

**Published:** 2023-11-07

**Authors:** Ismini Mpalatsouka, Myria Zachariou, Maria Kyprianidou, Georgia Fakonti, Konstantinos Giannakou

**Affiliations:** Department of Health Sciences, School of Sciences, European University Cyprus, Nicosia, Cyprus

**Keywords:** preeclampsia, cardiovascular disease, hypertensive disorders of pregnancy, women’s health, hypertension, Cyprus

## Abstract

Pregnancy complications, such as hypertensive disorders, present a substantial global public health challenge, with significant long-term implications for maternal and offspring health. This cross-sectional study aims to determine the level of awareness regarding long-term health risks among women who experienced preeclampsia during pregnancy in Cyprus and Greece. The study participants included adult women with a history of preeclampsia, while women with normal pregnancies were used as the comparison group. Data collection took place between June 2021 and February 2022, utilizing an online, self-administered questionnaire. The study included 355 women, with 139 (39.2%) in the preeclampsia group and 216 (60.8%) in the comparison group. Findings revealed that more than half of the women with prior preeclampsia (55.4%) were not aware of hypertensive disorders that can occur during pregnancy before their diagnosis, and a similar percentage (45.2%) had not received information about the long-term health risks following their diagnosis. Remarkably, only 3 participants (4.7%) with a history of preeclampsia were aware of the risk of developing cardiovascular diseases. There were no statistically significant differences between the preeclampsia and the comparison group regarding their concerns about long-term health risks, frequency of health checks, perceptions of factors influencing cardiovascular disease development, and doctor communication about different health topics, except from hypertension or high blood pressure. The study underscores the low level of awareness of long-term health risks among women with prior preeclampsia in Cyprus and Greece. This emphasizes the importance of implementing public health programs aimed at promoting cardiovascular risk assessment and effective management, both for clinicians and women with have experienced preeclampsia.

## Introduction

Pregnancy complications are a crucial public health challenge with a considerable and increasing prevalence worldwide (1–3). Hypertensive disorders of pregnancy (chronic hypertension, gestational hypertension, chronic hypertension with superimposed preeclampsia, preeclampsia, and eclampsia) are primary causes of maternal and offspring morbidity and mortality (4–8), and it was previously estimated that they affect around 10% of pregnancies globally (8). Preeclampsia is the most common hypertensive disorder of pregnancy, characterized by sudden onset of high blood pressure, usually after 20 weeks of gestation, and frequently accompanied by high protein levels in the urine (9, 10). Despite extensive research to determine the cause of preeclampsia, the exact underlying pathophysiology remains complex and unknown, with several environmental, genetic, and maternal factors all playing a role (11). Current evidence shows that no single test predicts pre-eclampsia with sufficient accuracy to be clinically useful, but a combination of markers into multiparametric models may provide a more useful and feasible predictive tool for preeclampsia screening in the routine care setting than a test of either component alone (12–15).

Over the past decades, a considerable amount of literature has been published on pregnancy complications and long-term health risks for both mother and offspring (6). Women with a history of hypertension during pregnancy have an increased risk of future hypertension, stroke, and cardiovascular disease (CVD) (16, 17). Also, offspring from pregnancies complicated by hypertensive disorders are small for gestational age and have an increased risk of CVD in adulthood (8, 18). With the increased incidence of physical and behavioral health conditions such as obesity, CVD, diabetes, anxiety, and depression, more pregnancies are expected to be complicated by pre-existing maternal conditions (19, 20). Of particular concern is the increased prevalence of CVD, which remains the leading cause of mortality in women worldwide with approximately 18 million deaths annually (21, 22).

Inadequate preventable measurements, such as women’s health awareness, may influence the incidence of hypertensive disorders, because women with a high level of awareness are more likely to report signs and symptoms, resulting in more efficient healthcare (23, 24). Different levels of awareness have been observed among women, with sociodemographic factors such as occupation and educational level being associated with poor awareness (25). Currently, there are no data on the prevalence of hypertensive disorders of pregnancy and women’s awareness of long-term health risks after experiencing hypertension during pregnancy in Cyprus and Greece. Therefore, this study attempts to reveal the level of awareness of long-term health risks among women who experienced preeclampsia during pregnancy in Cyprus and Greece.

## Methods

### Study design

This was a cross-sectional survey, following the Strengthening the Reporting of Observational Studies in Epidemiology guidelines (26).

### Study duration and study population

This study was conducted from June 2021 to February 2022. The population of interest included Greek and Greek-Cypriot women aged 18 years and older, living in the five government-controlled municipalities of the Republic of Cyprus or in any geographical area of Greece with a medical history of preeclampsia. Also, women with normal pregnancies were eligible to participate to serve as a comparison group. Women with multiple pregnancies or those who experienced unsuccessful pregnancy outcomes such as stillbirth, neonatal death, or preterm delivery were excluded to maintain the homogeneity of the study population.

### Sample recruitment and data collection

The questionnaire was administered using Google Forms and disseminated using social media applications (e.g., Facebook, Instagram), instant messaging apps (e.g., WhatsApp, Viber), and social networking sites (e.g., LinkedIn) to gather a sample from all geographical areas of Greece and Cyprus. A convenience sampling method was used, with a potential effect on sampling possibilities because of the quarantine restrictions resulting from the COVID-19 pandemic.

### Questionnaire

A self-administered online questionnaire in the Greek language was used for data collection, which it was specially created by the authors after an extensive review of relevant literature (Supplementary File S1). The survey instrument was pilot tested by 20 women for face validity and comprehensibility before study commencement. The sample of the pilot study was not included in the analysis.

The first part of the questionnaire included questions, based on a 194 validated questionnaire (27), to a participants’ pregnancy outcome status (preeclampsia vs. comparison group). In particular, participants were asked: (i) if they have been diagnosed with hypertension before pregnancy by a physician (possible answers: Yes, No, I am not sure); (ii) if during any pregnancy (lasting more than 6 months) their doctor diagnosed them with hypertension or high blood pressure; (ii) if during any pregnancy in which they had high blood pressure, they had (a) high levels of protein in the urine, (b) seizures or convulsions, (c) preeclampsia, eclampsia or toxemia; and (iv) if they had preeclampsia, eclampsia, or toxemia during any pregnancy. The second section of the questionnaire examined participants’ socio-demographic characteristics, while, the third section of the questionnaire assessed participants’ concerns about long-term health risks, as well as the frequency of participant-doctor communication and participation in health checks. The fourth section of the questionnaire evaluated women’s knowledge and perceptions of hypertension disorders during pregnancy. The final section of the questionnaire asked whether women were informed about hypertensive disorders of pregnancy before or after their pregnancies, as well as potential long-term health risks. This section also included questions about how they were informed and the preventive measures they undertook.

### Statistical analysis

The distribution of the continuous variables was examined using the Shapiro–Wilk normality test. Continuous variables with skewed distributions summarized as median and interquartile range (IQR). Absolute (n) and relative (%) frequency were used to report categorical variables. The chi-square test of independence was used to evaluate any association between preeclampsia and comparison groups and the categorical characteristics of the participants. Preeclampsia status was coded as a binary variable (preeclampsia group vs. comparison group), and it served as the main outcome of the study. Moreover, a sensitivity analysis was conducted to examine the influence of two distinct time intervals: respondents diagnosed with preeclampsia within 3 years of this study’s conduction (≤ 3 years) and those diagnosed more than 3 years ago (> 3 years). The chi-square test of independence was used to assess associations between the time since study conduction and sociodemographic characteristics, participants’ concerns about long-term health risks, participant-doctor communication, and health checks. All statistical tests were two-sided with a statistical significance level of *α* = 0.05. Statistical analysis was conducted using STATA 14.0 (Stata Corp, College Station, TX, United States).

### Ethics approval

The Cyprus National Bioethics Committee (CNBC) (ΕΕΒΚ-ΕΠ 2021.01.137) approved the study. All participants were informed about the purpose of the study and that participation was voluntary and anonymous. The respondents needed to confirm their willingness to participate voluntarily by answering a “Yes” or “No” question on a written informed consent form before being allowed to complete the online self-reporting questionnaire.

## Results

### Participants’ characteristics

A total of 355 women participated in the study with the median age being 36 years old (1st quartile = 31, 3rd quartile = 39). More than half of the participants were from Greece (*n* = 191, 54.1%) and almost 45% (*n* = 158) were from Cyprus. Most of the participants were from the capital of Cyprus, Nicosia (*n* = 81, 25.3%) and from the capital of Greece, Athens (*n* = 75, 23.4%). Approximately 78% (*n* = 275) of the respondents were residents of urban regions. In addition, most of the participants had completed an undergraduate education (*n* = 183, 52.0%) without proceeding to postgraduate studies, were Christian Orthodox (*n* = 336, 96.5%), were married/in cohabitation (*n* = 335, 94.4%), were private employees (*n* = 165, 46.7%), and had a monthly income from 501 to 1,000 euros (*n* = 114, 32.7%) (Table 1).

**Table 1:** Sociodemographic characteristics of all responders.

**Characteristics**	**Overall** (*N* = 355)
**Age**, Median (IQR)	36 (31, 39)
**Country**, *N*^a^ (%)	
Cyprus	158 (44.8)
Greece	191 (54.1)
Other	4 (1.1)
**Geographical area**, *N*^b^ (%)	
Nicosia	81 (25.3)
Athens	75 (23.4)
Limassol	54 (16.9)
Other in Greece	51 (15.9)
Larnaca	24 (7.5)
Thessaloniki	18 (5.6)
Other	7 (2.2)
Paphos	5 (1.6)
Ammochostos	5 (1.6)
**Residency**, *N*^c^ (%)	
Urban	275 (78.1)
Rural	77 (21.9)
**Educational level**, *N*^c^ (%)	
Secondary education	47 (13.3)
Undergraduate education	183 (52.0)
Postgraduate education	122 (34.7)
**Religion**, *N*^d^ (%)	
Christian Orthodox	336 (96.5)
No religion/Other	12 (3.5)
**Marital status**, *N*^e^ (%)	
Married/In cohabitation	335 (94.4)
Unmarried	7 (2.0)
Divorced/separated/widowed	13 (3.6)
**Occupation**, *N*^a^ (%)	
Private employee	165 (46.7)
State employee	80 (22.7)
Freelancer	41 (11.6)
Unemployed	35 (9.9)
Housewife	28 (7.9)
Retired	4 (1.2)
**Monthly income**, *N*^f^ (%)	
No income	46 (13.2)
Less than €500	18 (5.2)
€501- €1,000	114 (32.7)
€1,001- €1,501	78 (22.3)
€1,501- €2000	44 (12.6)
More than €2001	49 (14.0)

IQR; interquartile range; ^a^*N* = 353; ^b^*N* = 320; ^c^*N* = 352; ^d^*N* = 348; ^e^*N* = 355; ^f^*N* = 349.

### Concerns about long-term health risks, doctor communication, and health checks

We included 139 (39.2%) women in the preeclampsia group and 216 (60.8%) in the comparison group. Out of the 139 women with a history of preeclampsia, the largest proportion reported having comorbidities such as hypertension (22.6%), increased BMI (14.1%), diabetes (11.3%), thyroid diseases (1.1%) and kidney diseases (1.1%), while only 28.3% reported no health issue. A large proportion of participants (both preeclampsia and comparison group) expressed a little concern about having a risk of developing CVD (*n* = 118, 35.3%), cancer (excluding breast cancer) (*n* = 126, 39.0%), breast cancer (*n* = 129, 40.2%) and hypertension/high blood pressure (*n* = 103, 30.6%). Conversely, a substantial proportion of participants had no concerns about being at risk of weight problems/obesity (*n* = 119, 35.1%), osteoporosis (*n* = 150, 46.4%), diabetes (*n* = 136, 42.6%), or dementia or Alzheimer’s disease (*n* = 150, 46.4%). Overall, we did not identify any statistically significant difference between the preeclampsia and comparison group regarding their concerns about long-term health risks (Table 2).

**Table 2:** Information on participants’ concerns about long-term health risks, frequency of participant-doctor communication, and health checks in preeclampsia and comparison group.

Characteristics	Overall (*N* = 355)	Preeclampsia group (*N* = 139)	Comparison group (*N* = 216)	*p*-value^o^
**To what extent are you concerned that you may be at risk of the following diseases?**				
**Cardiovascular diseases**, *N*^a^ (%)				
Not at all	95 (28.4)	41 (31.0)	52 (26.7)	0.463
Little	118 (35.3)	51 (38.3)	65 (33.3)	
Enough	85 (25.5)	27 (20.3)	57 (29.2)	
Very	25 (7.5)	10 (7.5)	14 (7.2)	
Extremely	11 (3.3)	4 (2.9)	7 (3.6)	
**Weight problems/Obesity**, *N*^b^ (%)				
Not at all	119 (35.1)	54 (40.6)	63 (31.5)	0.357
Little	86 (25.4)	32 (24.1)	53 (26.5)	
Enough	91 (26.8)	35 (26.3)	54 (27.0)	
Very	21 (6.2)	6 (4.5)	14 (7.0)	
Extremely	22 (6.5)	6 (4.5)	16 (8.0)	
**Cancer (except breast cancer)**, *N*^c^ (%)				
Not at all	110 (34.1)	44 (34.4)	63 (33.3)	0.627
Little	126 (39.0)	48 (37.5)	76 (40.2)	
Enough	67 (20.7)	30 (23.4)	36 (19.1)	
Very	10 (3.1)	2 (1.6)	8 (4.2)	
Extremely	10 (3.1)	4 (3.1)	6 (3.2)	
**Breast cancer**, *N*^d^ (%)				
Not at all	107 (33.3)	45 (36.0)	59 (31.0)	0.112
Little	129 (40.2)	41 (32.8)	85 (44.7)	
Enough	60 (18.7)	31 (24.8)	29 (15.3)	
Very	15 (4.7)	5 (4.0)	10 (5.3)	
Extremely	10 (3.1)	3 (2.4)	7 (3.7)	
**Hypertension/High blood pressure**, *N*^e^ (%)				
Not at all	90 (26.7)	30 (22.7)	58 (29.2)	0.788
Little	103 (30.6)	42 (31.8)	59 (29.7)	
Enough	86 (25.5)	36 (27.3)	49 (24.6)	
Very	35 (10.4)	14 (10.6)	20 (10.0)	
Extremely	23 (6.8)	10 (7.6)	13 (6.5)	
**Osteoporosis**, *N*^c^ (%)				
Not at all	150 (46.4)	60 (47.6)	88 (46.1)	0.820
Little	119 (36.8)	43 (34.1)	72 (37.7)	
Enough	42 (13.0)	18 (14.3)	24 (12.6)	
Very	9 (2.8)	3 (2.4)	6 (3.1)	
Extremely	3 (1.0)	2 (1.6)	1 (0.5)	
**Diabetes**, *N*^f^ (%)				
Not at all	136 (42.6)	54 (43.5)	79 (41.8)	0.816
Little	89 (27.9)	30 (24.2)	57 (30.2)	
Enough	62 (19.4)	26 (21.0)	36 (19.1)	
Very	19 (6.0)	8 (6.5)	10 (5.3)	
Extremely	13 (4.1)	6 (4.8)	7 (3.7)	
**Dementia/Alzheimer**, *N*^c^ (%)				
Not at all	150 (46.4)	60 (47.2)	88 (46.3)	0.783
Little	108 (33.4)	39 (30.7)	65 (34.2)	
Enough	49 (15.2)	21 (16.5)	28 (14.8)	
Very	7 (2.2)	2 (1.6)	5 (2.6)	
Extremely	9 (2.8)	5 (4.0)	4 (2.1)	
**How often do you talk to your doctor about any of the following?**				
**Cardiovascular diseases**, *N*^b^ (%)				
Not at all	202 (59.6)	81 (60.4)	119 (59.8)	0.943
Little	77 (22.7)	32 (23.9)	44 (22.1)	
Enough	44 (13.0)	16 (11.9)	25 (12.6)	
Very	12 (3.5)	4 (3.0)	8 (4.0)	
Extremely	4 (1.2)	1 (0.8)	3 (1.5)	
**Weight problems/Obesity**, *N*^e^ (%)				
Not at all	179 (53.1)	76 (58.0)	100 (50.0)	0.615
Little	92 (27.3)	33 (25.2)	58 (29.0)	
Enough	48 (14.2)	17 (13.0)	30 (15.0)	
Very	13 (3.9)	3 (2.3)	9 (4.5)	
Extremely	5 (1.5)	2 (1.5)	3 (1.5)	
**Cancer (except breast cancer)**, *N*^g^ (%)				
Not at all	238 (72.1)	94 (72.9)	140 (71.8)	0.594
Little	69 (20.9)	28 (21.7)	39 (20.0)	
Enough	15 (4.6)	6 (4.6)	9 (4.6)	
Very	6 (1.8)	1 (0.8)	5 (2.6)	
Extremely	2 (0.6)	0 (0.0)	2 (1.0)	
**Breast cancer**, *N*^h^ (%)				
Not at all	213 (65.5)	84 (65.6)	125 (65.5)	0.303
Little	76 (23.4)	34 (26.6)	40 (20.9)	
Enough	27 (8.3)	9 (7.0)	18 (9.4)	
Very	7 (2.2)	1 (0.8)	6 (3.1)	
Extremely	2 (0.6)	0 (0.0)	2 (1.1)	
**Hypertension/High blood pressure**, *N*^i^ (%)				
Not at all	160 (47.1)	52 (39.1)	104 (51.7)	**0.028**
Little	93 (27.3)	43 (32.3)	50 (24.9)	
Enough	59 (17.3)	30 (22.6)	28 (13.9)	
Very	20 (5.9)	4 (3.0)	15 (7.5)	
Extremely	8 (2.4)	4 (3.0)	4 (2.0)	
**Osteoporosis**, *N*^j^ (%)				
Not at all	268 (81.7)	103 (82.4)	160 (81.2)	0.656
Little	44 (13.4)	18 (14.4)	25 (12.7)	
Enough	13 (4.0)	4 (3.2)	9 (4.6)	
Very	2 (0.6)	0 (0.0)	2 (1.0)	
Extremely	1 (0.3)	0 (0.0)	1 (0.5)	
**Diabetes**, *N*^k^ (%)				
Not at all	228 (68.9)	91 (71.1)	133 (67.5)	0.184
Little	53 (16.0)	15 (11.7)	37 (18.8)	
Enough	32 (9.7)	15 (11.7)	17 (8.6)	
Very	10 (3.0)	2 (1.6)	7 (3.6)	
Extremely	8 (2.4)	5 (3.9)	3 (1.5)	
**Dementia/Alzheimer**, *N*^l^ (%)				
Not at all	286 (86.9)	113 (89.0)	168 (85.7)	0.848
Little	33 (10.0)	11 (8.6)	21 (10.7)	
Enough	7 (2.1)	2 (1.6)	5 (2.6)	
Very	3 (1.0)	1 (0.8)	2 (1.0)	
Extremely	0 (0.0)	0 (0.0)	0 (0.0)	
**How often do you check your blood pressure?** *N*^m^ (%)				
Never	27 (7.7)	12 (8.7)	15 (7.2)	0.121
1 time/year	33 (9.4)	8 (5.8)	25 (12.0)	
2–3 times/year	98 (27.8)	35 (25.4)	61 (29.3)	
1 time/month	84 (23.9)	32 (23.2)	50 (24.1)	
1 time/week	44 (12.5)	24 (17.4)	19 (9.1)	
More than 1 time/ week	66 (18.7)	27 (19.5)	38 (18.3)	
**How often do you check your glucose levels?** *N*^n^ (%)				
Never	99 (28.0)	37 (26.6)	62 (29.7)	0.560
1 time/year	133 (37.6)	47 (33.8)	82 (39.2)	
2–3 times/year	68 (19.2)	33 (23.7)	35 (16.7)	
1 time/month	24 (6.8)	10 (7.2)	12 (5.7)	
1 time/week	10 (2.8)	3 (2.2)	7 (3.4)	
More than 1 time/ week	20 (5.6)	9 (6.5)	11 (5.3)	

^a^*N* = 334; ^b^*N* = 339; ^c^*N* = 323; ^d^*N* = 321; ^e^*N* = 337; ^f^*N* = 319; ^g^*N* = 330; ^h^*N* = 325; ^i^*N* = 340; ^j^*N* = 328; ^k^*N* = 331; ^l^*N* = 329; ^m^*N* = 352; ^n^*N* = 354; ^o^Differences between preeclampsia group and comparison group were tested using chi^2^ test; Bold indicates statistically significant associations (*p* ≤ 0.05).

Furthermore, a majority of participants did not discuss various health topics with their doctor, including CVD (*n* = 202, 59.6%), weight problems/obesity (*n* = 179, 53.1%), cancer excluding breast cancer (*n* = 238, 72.1%), breast cancer (*n* = 213, 65.5%), hypertension/high blood pressure (*n* = 160, 47.1%), osteoporosis (*n* = 268, 81.7%), diabetes (*n* = 228, 68.9%) and dementia/Alzheimer’s disease (*n* = 286, 86.9%) (Table 2). Notably, the only statistically significant difference between the preeclampsia and comparison groups was found in terms of how often women consulted their doctor about hypertension or high blood pressure (*p* = 0.028). Specifically, in the preeclampsia group, almost 40% did not discuss hypertension/high blood pressure with their doctor (*n* = 52, 39.1%), while the corresponding percentage in comparison group is more than 50% (*n* = 104, 51.7%). In terms of monitoring their health, the majority checked their blood pressure either two to three times per year (*n* = 98, 27.8%) or once per month (*n* = 84, 23.9%). Nearly 38% (*n* = 133) of respondents checked their glucose levels once year, while 28.0% (*n* = 99) never checked their glucose levels. No statistically significant difference between the preeclampsia and comparison group was observed regarding how often they check their blood pressure or glucose levels (Table 2).

### Perceptions of factors influencing CVD development

Approximately 90% of the respondents (*n* = 317) attributed CVD to a combination of genetic predisposition (i.e., family history and genes) and lifestyle factors like exercise and diet. In contrast, 6.0% (*n* = 21) believed that CVD was solely due to genetic predisposition, while 4.2% (*n* = 15) thought it was solely due to lifestyle. Additionally, a majority of the participants either agreed (*n* = 195, 54.9%) or absolutely agreed (*n* = 127, 35.8%) that being overweight was a significant risk factor for developing CVD. However, no statistically significant differences were observed between the preeclampsia and comparison group regarding their perceptions of CVD development (Supplementary Table S1).

### Awareness of hypertensive disorders during pregnancy and long-term health risks among women with a history of preeclampsia

More than half of women with history of preeclampsia (*n* = 77, 55.4%) were not aware of hypertensive disorders that can occur during pregnancy before receiving their diagnosis. Among those who were aware about these disorders, the primary sources of information were from gynecologist/obstetrician (*n* = 31, 50%) and the internet (*n* = 14, 22.6%). Furthermore, 23.5% (*n* = 32) of women with a history of preeclampsia reported that healthcare professionals informed them about long-term health risks before childbirth and around 18.4% (*n* = 25) were informed after childbirth. The majority of participants with a history of preeclampsia received preventative treatment during pregnancy, with 45.3% taking medication (*n* = 63), and 32.4% (*n* = 45) adopting an improved diet. Approximately 22% (*n* = 31) did not receive any preventive treatment during pregnancy. The primary sources of information regarding these long-term health risks were gynecologist/obstetrician (*n* = 20, 62.5%), and the internet (*n* = 4, 12.5%). Among the long-term health risks, women reported awareness of developing hypertension (*n* = 48, 76.2%), diabetes (*n* = 9, 14.4%), CVD (*n* = 3, 4.7%), and kidney disease (*n* = 3, 4.7%) (Table 3).

**Table 3:** Awareness of hypertensive disorders during pregnancy and long-term health risks among women with a history of preeclampsia.

	**Preeclampsia group** (*N* = 139)
**Before your diagnosis were you aware of the hypertensive disorders that can occur during pregnancy?** *N*^a^ (%)	
No	77 (55.4)
Yes	62 (45.5)
**Source of information for hypertensive disorders that can occur during pregnancy**, *N*^b^ (%)	
Gynecologist/Obstetrician	31 (50.0)
Internet	14 (22.6)
Social environment	8 (12.9)
Literature/Books	4 (6.5)
Healthcare professional	3 (4.8)
No source	1 (1.6)
General Practitioner/Personal Physician	1 (1.6)
**At what time were healthcare professionals informed about the long-term health risks after your diagnosis?** *N*^c^ (%)	
Before childbirth	32 (23.5)
After childbirth	25 (18.4)
Up to six weeks after childbirth	6 (4.4)
Six weeks-six months after childbirth	2 (1.5)
Six months-one year after childbirth	3 (2.2)
In more than 1 year	3 (2.2)
I do not remember	65 (47.8)
**Have you received any preventive care during your pregnancy?** *N*^a^ (%)	
Medication	63 (45.3)
Improved nutrition/diet	45 (32.4)
No preventive treatment	31 (22.3)
**Have you been informed that after pregnancy with hypertensive disorders there may be long-term risks to your health?** *N*^b^ (%)	
Yes	32 (51.6)
No	28 (45.2)
I am not sure	2 (3.2)
**Source of information for long-term health risks**, *N*^d^ (%)	
Gynecologist/Obstetrician	20 (62.5)
Internet	4 (12.5)
Midwife	3 (9.4)
General Practitioner/Personal Physician	2 (6.3)
Social environment	2 (6.3)
Cardiologist	1 (3.1)
**Which long-term health risks are you aware of?** *N*^e^ (%)	
Hypertension	48 (76.2)
Diabetes	9 (14.4)
Cardiovascular diseases	3 (4.7)
Kidney disease	3 (4.7)

^a^*N* = 139; ^b^*N* = 62; ^c^*N* = 136; ^d^*N* = 32; ^e^*N* = 63 (Multiple answers).

### Cardiometabolic assessments and use of preventive measures among women with a history of preeclampsia

We observed that 33.8% (*n* = 105) of women with a history of preeclampsia underwent hypertension screening as a pre-symptomatic measure after their last pregnancy. The most commonly preventive measures for long-term health risks were an improved diet (*n* = 53, 21.7%), followed by regular health checks with a doctor (*n* = 37, 15.2%), exercise (*n* = 32, 13.1%), abstaining from smoking (*n* = 23, 9.4%), medication (*n* = 22, 9.0%), and limiting alcohol consumption (*n* = 21, 8.6%). In contrast, 23.0% (*n* = 56) reported that they did not receive any preventive measure. Among women who experienced preeclampsia during their pregnancy, the majority sought care from a cardiologist (*n* = 70, 35.0%) or a general practitioner/personal physician (*n* = 41, 20.5%) after childbirth. Furthermore, most of those who followed preventive measures related to long-term health risks initiated these measures immediately after childbirth (*n* = 61, 44.5%). In terms of discussions with healthcare providers, most of the women with a history of preeclampsia occasionally asked their doctor about their heart health (*n* = 43, 31.2%), while a smaller proportion inquired very frequently (*n* = 5, 3.6%). Additionally, 57.3% (*n* = 79) of the participants had a heart check within the last 12 months, and among them, 61.8% (*n* = 76) had discussed the results with their doctor (Table 4).

**Table 4:** Cardiometabolic assessments and use of preventive measures among women with a history of preeclampsia.

	**Preeclampsia group** (*N* = 139)
**Have you received any of the following pre-symptomatic screening since your last pregnancy in which you experienced preeclampsia?** *N*^a^ (%)	
Hypertension check	105 (33.8)
Cholesterol check	27 (8.7)
Glucose check	28 (9.0)
Encouragement to stop smoking	10 (3.2)
Encouragement for weight control	43 (13.8)
Advice to exercise frequently	45 (14.5)
Advice on healthy eating	53 (17.0)
**Do you follow any preventive measures related to long-term health risks?** *N*^b^ (%)	
Monitoring by a doctor/medical specialist	37 (15.2)
Improved diet	53 (21.7)
Exercise	32 (13.1)
Avoiding smoking	23 (9.4)
Medication	22 (9.0)
Avoiding high alcohol consumption	21 (8.6)
No preventive measure	56 (23.0)
**Given that you experienced hypertensive disorder problems (e.g., preeclampsia) during your pregnancy, did you go to any of the following after giving birth?** *N*^c^ (%)	
Cardiologist	70 (35.0)
General Practitioner / Personal Physician	41 (20.5)
I do not remember	30 (15.0)
Nutritionist	23 (11.5)
Nephrologist	22 (11.0)
Gym	7 (3.5)
Other^1^	7 (3.5)
**Time of preventive measures**, *N*^d^ (%)	
Right after childbirth	61 (44.5)
3–12 months after childbirth	12 (8.8)
1–3 years after childbirth	7 (5.1)
4–6 years after childbirth	3 (2.2)
Never	54 (39.4)
**How often do you ask your doctor about your heart health?** *N*^e^ (%)	
I do not visit a doctor	14 (10.1)
Never	21 (15.2)
Rarely	40 (29.0)
Sometimes	43 (31.2)
Often	15 (10.9)
Very often	5 (3.6)
**Heart check in the last 12 months**, *N*^e^ (%)	
Yes	79 (57.3)
No	58 (42.0)
I am not sure	1 (0.7)
**If you had a heart check in the last 12 months, have you discussed the results with your doctor?** *N*^f^ (%)	
Yes	76 (61.8)
No	44 (35.8)
I am not sure	3 (2.4)

^a^*N* = 311 (Multiple answers); ^b^*N* = 244 (Multiple answers); ^c^*N* = 200 (Multiple answers); ^d^*N* = 137; ^e^*N* = 138; ^f^*N* = 123; ^1^Other: Endocrinologist, Rheumatologist, Dialectologist, Ophthalmologist.

### Sensitivity analysis

We divided respondents into two distinct intervals based on their self-report recall of clinician diagnosis of preeclampsia: those diagnosed within the past 3 years (≤ 3 years) and those diagnosed more than 3 years ago (> 3 years). We did not find any statistically significant impact of time since the study’s conduction on participants’ responses regarding concerns about long-term health risks, participant-doctor communication, and health checks (all *p*-values >0.05). Therefore, the results of the sensitivity analysis were consistent with those in the main analysis (Supplementary File S2).

## Discussion

The aim of this study was to determine the level of awareness of long-term health risks among women in Cyprus and Greece who had preeclampsia during pregnancy. We included both women who experienced preeclampsia and women without history of preeclampsia as a comparison group. Our results indicate that more than 50% of women who had experienced preeclampsia were unaware of hypertensive disorders that can occur during pregnancy prior to their preeclampsia diagnosis, while a similar percentage had not been informed about the long-term health risks following their diagnosis. There were no statistically significant differences between the preeclampsia and the comparison group regarding their concerns about long-term health risks, frequency of health checks, perceptions of factors influencing CVD development, and doctor communication about different health topics except for hypertension or high blood pressure.

Our results show that women with a history of preeclampsia, and those with normal pregnancies, were not concerned about the development of long-term health conditions such as CVD, obesity, hypertension, and others. This might be associated with the lack of knowledge about the link of preeclampsia with long-term health complications as we observed in our study. Previous research also showed that women with prior preeclampsia had little or no knowledge about the link between hypertensive disorders during pregnancy and CVD, neither after delivery nor in the long-term post-partum period (28–30). However, in our study, we did not specifically address any concerns regarding the health status of their offspring following a diagnosis of preeclampsia. This is noteworthy as previous research indicates that women with preeclampsia tend to be more concerned about the health of their offspring instead of their own health (31, 32).

As part of our study, we explored patient-doctor communication and monitoring of glucose and blood pressure. Of note, we discovered that women with a history of preeclampsia seemed to discuss hypertension more frequently than women with normal pregnancies. This could be because high blood pressure is a common symptom of preeclampsia, which could raise concerns in this situation. Regarding the frequency of blood pressure and blood sugar levels checks, the majority of participants said they checked their blood pressure 2–3 times a year, while most of women said they checked their glucose levels once a year. Interestingly, we did not find any difference in frequency of those checks between women with history of preeclampsia and normal pregnancies. According to our findings, a previous study found that women with a history of preeclampsia did not have blood pressure checks after pregnancy, while glucose and cholesterol checks were uncommon and similar in women with and without preeclampsia (33).

The perceptions of factors influencing CVD development and the primary sources of information of long-term health risks were also assessed. A combination of genetic and lifestyle factors was the most common answer to the question about, while only a very small percentage of participants, irrespective of preeclampsia history, believed only at the influence of genetic factors. Women’s skepticism regarding the sole influence of genetic predisposition on the development of CVD suggest a hopeful outlook for interventions targeting lifestyle changes; nevertheless, implementing such alterations can be challenging even for those who have a genetic risk (34). Regarding the information about the long-term health risks after a history of preeclampsia, we identified that more than half with preeclampsia history did not receive any information. Among those who received some information the primary source of information was their gynecologist/obstetrician.

In our study, we observed that women with a history of preeclampsia were less likely to engage in discussions about their health with their doctors. Although the majority underwent hypertension screening as a precautionary measure following their pregnancy, most did not adopt long-term preventative measures to mitigate potential health risks. Earlier research found that women with a history of preeclampsia did not receive any counseling for making healthy lifestyle changes, following up after their diagnosis, or talking to their doctor about potential preeclampsia risks in the future, which is similar to our findings (30, 31). Moreover, women with a history of preeclampsia were asked about their preventive measures they had been followed after their last pregnancy. A third of the participants said they had only checked their blood pressure; 23% said they had not received any preventive measures related to long-term health risks; and only a small percentage said they had received preventive measures such as a healthy diet and exercise. In addition, 35% of them reported visiting a cardiologist after their pregnancy complicated with preeclampsia. In accordance with our findings, a previous study that examined lifestyle modifications and cardiovascular risk assessment in women with a previous preeclampsia found that most of the study population was aware of the probability of developing hypertension; however, the majority was not sensitized to adopt a healthier lifestyle, and in some cases, they even implemented a worse lifestyle (35).

Our findings have several public health and clinical implications. Our findings show that while women with prior preeclampsia have some basic knowledge about long-term health risks, this knowledge is often superficial and incomplete. This lack of information about the possible long-term health risks is undoubtedly an important public health issue considering that hypertensive disorders during pregnancy, particularly preeclampsia, increases the risk of women developing CVD (6). Previous studies that assessed the knowledge among healthcare providers (e.g., physicians, obstetricians, gynecologists) on the association between preeclampsia and maternal long-term adverse outcomes as well as whether they were providing appropriate counseling and care to these women show that in most cases their knowledge is adequate or lacking (36–38). Furthermore, there is inconsistency in the existing guidelines for the long-term treatment of these patients as well as in the their follow up care and counseling (36–38). Continuous medical education and other educational activities, such as presentations and seminars on the efficient implementation of follow-up care and counseling, are required, emphasizing the importance of counseling on lifestyle modifications, such as increased physical activity and weight management, as well as timely cardiovascular screening and diagnostic tests for women with prior preeclampsia. Previous research indicates that these women are eager to learn and be informed (39). Thus, more detailed education about long-term health risks and participation in decisions about ongoing care for women with prior preeclampsia should be encouraged, as this may increase compliance and, as a result, improve their outcome. Further research is necessary to determine an evidence-based guideline for the follow-up of women with prior preeclampsia.

Despite the great importance of our study, some limitations should be considered in interpreting the findings. Firstly, this is a cross-sectional study, and as such, it cannot establish a causal relationship. Secondly, data collection was done using a convenient online survey, which may limit the representativeness of our study. A limitation of our study is the presence of some missing data that might impact on the completeness and accuracy of our results. Moreover, a history of preeclampsia was obtained through self-report recall of clinician diagnosis, introducing the possibility of recall bias. Also, excluding women with multiple pregnancies or unsuccessful outcomes may limit the generalizability of our findings to a broader population of women with diverse pregnancy outcomes. Furthermore, our questionnaire did not capture specific details related to the onset of hypertension, the severity of preeclampsia, and the types of antihypertensive medications used. These aspects are crucial for a comprehensive understanding of preeclampsia and its management. Future studies should endeavor to include these specific aspects in their data collection to enhance the depth of knowledge in this area.

## Conclusion

Our findings show that over half of the women with a history of preeclampsia in Cyprus and Greece were unaware of hypertensive disorders during pregnancy before their preeclampsia diagnosis, and a similar percentage had not received information about the long-term health risks following their diagnosis. No statistically significant differences were observed between the preeclampsia and comparison groups concerning their concerns about long-term health risks, the frequency of health examinations, perceptions of factors influencing CVD development, and doctor-patient communication on various health matters, except for hypertension. It is critical to develop evidence-based programs to encourage women to make lifestyle changes and improve their knowledge of and adherence to recommended follow-up and monitoring, as well as to encourage clinicians to consider cardiovascular risk assessment and active management in women with a history of preeclampsia.

## Data availability statement

The raw data supporting the conclusions of this article will be made available by the authors, without undue reservation.

## Ethics statement

The studies involving humans were approved by the Cyprus National Bioethics Committee (ΕΕΒΚ-ΕΠ 2021.01.137). The study were conducted in accordance with the local legislation and institutional requirements. The participants provided their electronic informed consent to participate in this study.

## Author contributions

IM: investigation, methodology, writing—original draft. MZ: investigation, methodology, writing—review and editing. MK: formal analysis, methodology, visualization, writing—original draft. GF: writing—original draft. KG: conceptualization, investigation, formal analysis, methodology, project administration, supervision, writing—original draft. All authors read and approved the final manuscript.
