# Supplementary material for: Assessing awareness of long-term health risks among women with a history of preeclampsia: a cross-sectional study
Source: Front Med (Lausanne). 2023 Nov 7;10:1236314. doi: 10.3389/fmed.2023.1236314 (PMC10662303; doi:10.3389/fmed.2023.1236314)
Supplement: Supplementary file 1 [file Table_1.docx]

**Supplementary Table 1.** Preeclampsia and comparison group's perceptions about of factors influencing cardiovascular disease development .

| **Characteristics** | **Overall**  (N=355) | **Preeclampsia group** (N=139) | **Comparison group**  (N=210) | **p-value**^c^ |
| --- | --- | --- | --- | --- |
| **In your opinion, cardiovascular diseases are due to:** N^a^ (%) | | | | |
| Genetic predisposition (family history, genes) | 15 (4.2) | 9 (6.6) | 6 (2.9) | 0.238 |
| Lifestyle (e.g., exercise, diet) | 21 (6.0) | 8 (5.8) | 11 (5.2) |  |
| Both | 317 (89.8) | 120 (87.6) | 193 (91.9) |  |
| **Is being overweight an important risk factor for developing cardiovascular disease?** N^b^ (%) | | | | |
| Absolutely disagree | 1 (0.3) | 1 (0.7) | 0 (0.0) | 0.584 |
| Disagree | 1 (0.3) | 0 (0.0) | 1 (0.5) |  |
| Neither agree nor disagree | 31 (8.7) | 12 (8.6) | 19 (9.1) |  |
| Agree | 195 (54.9) | 80 (57.6) | 112 (53.3) |  |
| Absolutely agree | 127 (35.8) | 46 (33.1) | 78 (37.1) |  |
| ^a^N=353; ^b^N=355; ^c^Differences between preeclampsia group and comparison group were tested using chi^2^ test. | | | | |
