# Supplementary material for: Assessing awareness of long-term health risks among women with a history of preeclampsia: a cross-sectional study
Source: Front Med (Lausanne). 2023 Nov 7;10:1236314. doi: 10.3389/fmed.2023.1236314 (PMC10662303; doi:10.3389/fmed.2023.1236314)
Supplement: Supplementary file 2 [file Data_Sheet_1.docx]

**Supplementary S1:** **Electronic consent form and questionnaire in Greek and translated to English. (The original form in Greek is provided below).**

**LONG-TERM RISKS TO WOMEN'S HEALTH AFTER HYPERTENSIVE DISORDERS OF PREGNANCY**

***What is the purpose of this research?***

This anonymous questionnaire aims to record adult women's level of awareness and knowledge of long-term health risks following the presence of hypertensive disorders (e.g., pre-eclampsia) during pregnancy. The purpose of the survey is purely scientific. The study is aimed at adult women of all age groups who were diagnosed with gestational hypertensive disorders (e.g., pre-eclampsia) during pregnancy and are resident in Cyprus.

***Important information***

Participation is anonymous and voluntary, and the time required to complete the questionnaire ranges from 8 to 10 minutes. Please select the answer that represents your opinion. Please answer the following questions as honestly as possible. All information will be kept strictly confidential and will not be disclosed to any other person, except for the survey staff. All information will be collected, coded, and destroyed at the end of the survey. The research is being conducted by European University Cyprus and has been approved by the National Bioethics Committee of Cyprus (ΕΕΒΚ ΕΠ 2021.01.137). If you have any questions about the research, you can email the study coordinator, Dr. Konstantinos Giannakou (K.Giannakou@euc.ac.cy).

1. In order to complete the questionnaire, please confirm that you have read and understood the above privacy agreement and agree that your data may be used anonymously for statistical analysis and scientific publication.

 I have been informed about the privacy policy and I authorise the use of this questionnaire and my answers for anonymous statistical analysis and scientific publication

 I am not interested in participating in this survey.

1. Please indicate your age.

 I am under 18 years old (<18)

 I am 18 years old or older (≥18)

1. I declare that the information I provide is TRUE

 Yes

 No

**SECTION Α – DEMOGRAPHICS**

Α1. What is your age (in years)? ...........................

Α2. What is your country of origin (your nationality)?

1. Cyprus □

2. Greece □

3. Other □ (Specify): ........................

Α3. What is your region of residence?

1. Nicosia □

2. Limassol □

3. Paphos □

4. Larnaca □

5. Ammochostos □

6. Other □ (Specify): ........................

A4. Please indicate whether you live in an urban or rural area.

1. Urban area (city)

2. Rural area (village)

A5. What is your highest level of education?

1. Primary school □

2. High school □

3. College □

4. University/Degree □

5. Postgraduate degree □

6. PhD □

A6. What is your religion?

1. Christian Orthodox □

2. Christian Catholic □

3. Jehovah's Witness □

4. Other □ (Specify): ..........................

A7. What is your marital status?

1. Single □

2. In cohabitation/relationship □

3. Married □

4. Separated □

5. Divorced □

6. Widowed □

Α8. Please indicate your professional status.

1. Private employee □

2. State employee □

3. Freelancer □

4. Unemployed □

5. Household □

6. Retired □

7. Other: □ (Specify): ..........................

Α9. Your monthly income is (EUR):

1. I have no income □

2. Less than 500 □

3. 501-1000 □

4. 1001-1500 □

5. 1501-2000 □

6. More than 2001 □

**SECTION Β - PREGNANCY HISTORY**

Β1. Are you pregnant at this time?

1. Yes □

2. No □

3. I am not sure □

Β2. Have you had at least one pregnancy that lasted longer than 6 months?

1. Yes □

2. No □

Β3. How many children do you have?

1. 0 □

2. 1 □

3. 2 □

4. 3 □

5. 4 or more □

Β4. Have you had high blood pressure in the past (before pregnancy)?

1. Yes □

2. No □

3. I am not sure □

Β5. Have you been diagnosed (before pregnancy) by a doctor with hypertension?

1. Yes □

2. No □

3. I am not sure □

Β6. During any pregnancy (lasting more than 6 months), did your doctor ever mention to you that you had high blood pressure or hypertension?

1. Yes, only in the first pregnancy □

2. No, in the first pregnancy, but in a subsequent pregnancy □

3. In the first pregnancy and at least one subsequent pregnancy □

4. No, in no pregnancy □

Β7. During any pregnancy in which you experienced elevated blood pressure, did you:

1. High protein levels in the urine □

2. Seizures or convulsions □

3. Pre-eclampsia, eclampsia, or toxaemia of pregnancy □

Β8. During any pregnancy did you experience preeclampsia, eclampsia, or toxemia?

1. Yes □

2. No □

Β9. How old were you when you first experienced pregnancy-related high blood pressure?

1. Age .................

**SECTION C - QUESTIONS RELATING TO GENERAL HEALTH**

C1. Have you ever had any of the following during, before or after pregnancy?

1. High blood pressure □

2. Diabetes □

3. High Body Mass Index (Obesity) □

4. Stroke □

5. Cardiovascular event □

6. Kidney problems □

7. None of the above / no major health problems □

8. Other □ (Specify): ................................

C2. From the list below, what currently applies to you (select all that apply to you)?

1. Smoking □

2. Obesity □

3. High alcohol consumption □

4. Sedentary life / Lack of physical activity □

5. Stressful work □

6. Rare or no consumption of fresh fruits and vegetables □

7. Family history of cardiovascular disease □

8. High cholesterol □

9. High blood pressure □

10. Diabetes □

11. None of the above □

12. Other □ (Specify): ..................................

C3. To what extent are you concerned that you may be at risk of the following diseases?

|  | Not at all | Little | Enough | Very | Extremely |
| --- | --- | --- | --- | --- | --- |
| Cardiovascular diseases |  |  |  |  |  |
| Weight problems / Obesity |  |  |  |  |  |
| Cancer (except breast cancer) |  |  |  |  |  |
| Breast cancer |  |  |  |  |  |
| Hypertension / High blood pressure |  |  |  |  |  |
| Osteoporosis |  |  |  |  |  |
| Diabetes |  |  |  |  |  |
| Dementia and Alzheimer's disease |  |  |  |  |  |

C4. How often do you talk to your doctor about each of the following?

|  | Not at all | Little | Enough | Very | Extremely |
| --- | --- | --- | --- | --- | --- |
| Cardiovascular diseases |  |  |  |  |  |
| Weight problems / Obesity |  |  |  |  |  |
| Cancer (except breast cancer) |  |  |  |  |  |
| Breast cancer |  |  |  |  |  |
| Hypertension / High blood pressure |  |  |  |  |  |
| Osteoporosis |  |  |  |  |  |
| Diabetes |  |  |  |  |  |
| Dementia and Alzheimer's disease |  |  |  |  |  |

C5. How often do you check your blood pressure?

1. Never □

2. Once a year □

3. 2-3 times a year □

4. Once a month □

5. Once a week □

6. More than once a week □

C6. How often do you check your glucose levels?

1. Never □

2. Once a year □

3. 2-3 times a year □

4. Once a month □

5. Once a week □

6. More than once a week □

**SECTION D - QUESTIONS ON KNOWLEDGE AND PERCEPTIONS OF HYPERTENSIVE DISORDERS IN PREGNANCY**

D1. In your opinion, which of the following, if any, could increase a woman's chance of developing cardiovascular disease? Select all that apply.

| Be overweight/obese |  |
| --- | --- |
| Consume high amounts of alcohol |  |
| Have an unhealthy diet |  |
| Have a family history of cardiovascular disease |  |
| Have high blood pressure |  |
| Smoking |  |
| Have high cholesterol |  |
| Have little or no physical activity |  |
| Have diabetes |  |
| Have had complications during pregnancy related to hypertensive disorders, such as pre-eclampsia |  |
| Have autoimmune diseases, such as lupus or rheumatoid arthritis |  |
| Have/had early menopause |  |
| Have/had irregular menstruation |  |
| I am not sure |  |

D2. In your opinion, cardiovascular diseases are caused by:

| Genetic predisposition (family history) |  |
| --- | --- |
| Lifestyle (e.g., exercise, diet) |  |
| Both of the above |  |

D3. Is being overweight a major risk factor for the development of cardiovascular disease?

| Absolutely disagree |  |
| --- | --- |
| Disagree |  |
| Neither agree nor disagree |  |
| Agree |  |
| Absolutely agree |  |

**SECTION E – QUESTIONS CONCERNING INFORMATION ON HYPERTENSIVE DISORDERS IN PREGNANCY**

Ε1. Before your diagnosis, were you aware of the hypertensive disorders (e.g., pre-eclampsia) that can occur during pregnancy?

1. Yes □

2. No □

Ε2. How were you informed before your diagnosis?

1. General Practitioner / Personal Physician □

2. Gynaecologist / Obstetrician □

3. Internet □

4. Social environment □

5. Other □ (Specify): ..............................

6. None □

Ε3. When a healthcare provider informed you about the long-term health risks after your diagnosis?

1. Before childbirth □

2. Immediately after childbirth □

3. Within 6 weeks after delivery □

4. From 6 weeks to 6 months after childbirth □

5. From 6 months to 1 year after childbirth □

6. More than 1 year □

7. I don't remember □

Ε4. During your pregnancy, have you received any preventive treatment (e.g., mild exercise or avoiding smoking and alcohol)?

1. Medication □

2. Improved diet □

3. No preventive treatment □

Ε5. Have you been informed that there may be long-term health risks after pregnancy with hypertensive disorders (e.g., pre-eclampsia)?

1. Yes □

2. No □

3. I am not sure □

Ε6. How were you informed about any long-term health risks?

1. General Practitioner / Personal Physician □

2. Gynaecologist / Obstetrician □

3. Midwife □

4. Internet □

5. Social environment □

6. Other □ (Specify): ..............................

7. None □

Ε7. What are the long-term health risks that you are aware of?

1. Hypertension □

2. Cardiovascular diseases □

3. Kidney disease □

4. Diabetes □

5. Other □ (Specify): ..............................

6. I don't know any □

Ε8. Have you received any of the following pre-symptomatic screening advice since your last pregnancy in which you experienced pre-eclampsia?

1. Checking arterial hypertension □

2. Cholesterol control □

3. Glucose control □

4. Encouragement to stop smoking □

5. Encouragement to control your weight □

6. Advice to exercise frequently □

7. Advice on healthy eating □

Ε9. Do you follow any preventive action related to long-term health risks?

1. Monitoring by a medical specialist □

2. Improved diet □

3. Exercise

4. Avoiding smoking

5. Avoiding high alcohol consumption

6. Medication □

7. No preventive action □

8. Other □ (Specify): ..............................

Ε10. Given that you experienced hypertensive disorder problems (e.g., preeclampsia) during your pregnancy, did you seek care from any of the following after delivery? Select all that apply.

1. Cardiologist □

2. Nephrologist □

3. General Practitioner / Personal Physician □

4. Gym □

5. Nutritionist □

6. I don't remember □

7. Other □ (Specify): ...................................

Ε11. When did you start taking prevention measures?

1. Immediately after childbirth □

2. 3 to 12 months after childbirth □

3. 1 to 3 years after childbirth □

4. 4-6 years after childbirth □

5. Never □

Ε12. How often do you ask your doctor about your heart health?

1. I do not visit a doctor □

2. Never □

3. Rarely □

4. Sometimes □

5. Often □

6. Very often □

Ε13. In the last 12 months have you had your heart checked by a doctor?

1. Yes □

2. No □

3. I am not sure □

Ε14. If you have had your heart checked in the last 12 months, have you discussed the results of the test with your doctor?

1. Yes □

2. No □

3. I am not sure □

**END OF QUESTIONNAIRE**

**Thank you very much for your time and your significant help in implementing this study.**

**ΕΡΩΤΗΜΑΤΟΛΟΓΙΟ**

**ΜΕΛΛΟΝΤΙΚΟΙ ΚΙΝΔΥΝΟΙ ΣΤΗΝ ΥΓΕΙΑ ΓΥΝΑΙΚΩΝ ΜΕΤΑ ΑΠΟ ΥΠΕΡΤΑΣΙΚΕΣ ΔΙΑΤΑΡΑΧΕΣ ΚΥΗΣΗΣ**

**Ποιος είναι ο σκοπός της έρευνας;**

Το παρόν ανώνυμο ερωτηματολόγιο έχει σκοπό την καταγραφή του επιπέδου ενημέρωσης και γνώσεων των ενήλικων γυναικών για τους μελλοντικούς κινδύνους στην υγεία τους μετά από την παρουσία υπερτασικών διαταραχών (π.χ. προεκλαμψία) κατά την διάρκεια της εγκυμοσύνης. Ο σκοπός της έρευνας είναι αποκλειστικά επιστημονικός. Η μελέτη απευθύνεται σε ενήλικες γυναίκες όλων των ηλικιακών ομάδων, οι οποίες κατά τη διάρκεια της εγκυμοσύνης τους διαγνωστήκαν με υπερτασικές διαταραχές κύησης (π.χ. προεκλαμψία) και είναι μόνιμοι κάτοικοι Κύπρου.

**Σημαντικές πληροφορίες**

Η συμμετοχή είναι ανώνυμη και εθελοντική και ο απαιτούμενος χρόνος για τη συμπλήρωση του ερωτηματολογίου κυμαίνεται από 8 έως 10 λεπτά. Παρακαλώ επιλέξτε την απάντηση που αντιπροσωπεύει την άποψή σας. Απαντήστε στις ακόλουθες ερωτήσεις όσο το δυνατόν πιο ειλικρινά. Όλες οι πληροφορίες θα παραμείνουν αυστηρά εμπιστευτικές και δεν θα γίνουν γνωστές σε κανένα άλλο άτομο, εκτός από το προσωπικό της έρευνας. Όλες οι πληροφορίες που θα συλλεχθούν, θα κωδικοποιηθούν, και θα καταστραφούν μετά το τέλος της έρευνας. Η έρευνα διενεργείται από το Ευρωπαϊκό Πανεπιστήμιο Κύπρου και έχει εγκριθεί από την Εθνική Επιτροπή Βιοηθικής Κύπρου (ΕΕΒΚ ΕΠ 2021.01.137). Αν έχετε οποιεσδήποτε ερωτήσεις για την έρευνα, μπορείτε να στείλετε email στον υπεύθυνο συντονιστή της μελέτης, Δρ. Κωνσταντίνο Γιαννακού (K.Giannakou@euc.ac.cy).

1. **Για να συμπληρώσετε το ερωτηματολόγιο, παρακαλώ επιβεβαιώστε ότι διαβάσατε και κατανοήσατε το ανωτέρω συμφωνητικό προστασίας προσωπικών δεδομένων και συμφωνείτε να γίνει χρήση ανωνύμως των στοιχείων σας για στατιστική ανάλυση και επιστημονική έκδοση;**

 Έχω πληροφορηθεί για την πολιτική προστασίας προσωπικών δεδομένων και επιτρέπω τη χρήση του ερωτηματολογίου αυτού και των απαντήσεών μου για ανώνυμη στατιστική ανάλυση και επιστημονική έκδοση

 Δεν ενδιαφέρομαι να συμμετάσχω στην έρευνα αυτή.

1. **Παρακαλώ δηλώστε την ηλικία σας.**

 Είμαι κάτω από 18 ετών (<18)

 Είμαι 18 ετών και άνω (≥18)

1. **Δηλώνω ότι οι πληροφορίες που θα δώσω είναι ΑΛΗΘΕΙΣ**

 Ναι

 Όχι

**Α. ΔΗΜΟΓΡΑΦΙΚΑ ΣΤΟΙΧΕΙΑ:**

**Α1. Ποια είναι η ηλικία σας (σε έτη);** ………………………

**Α2. Ποια είναι η χώρα καταγωγής σας (υπηκοότητα σας);**

1. Κύπρος □
2. Ελλάδα □
3. Άλλο □ (Προσδιορίστε): ……………..…….

**Α3. Ποια είναι η περιοχή της κατοικίας σας;**

1. Λευκωσία □
2. Λεμεσός □
3. Πάφος □
4. Λάρνακα □
5. Αμμόχωστος □
6. Άλλο □ (Προσδιορίστε): ……………..…….

**A4. Παρακαλώ δηλώστε εάν κατοικείτε σε αστική ή αγροτική περιοχή.**

1. Αστική περιοχή (πόλη)
2. Αγροτική περιοχή (χωριό)

**A5. Ποιο είναι το ανώτατο επίπεδο μόρφωσης σας;**

1. Δημοτικό □
2. Γυμνάσιο/Λύκειο □
3. Κολλέγιο □
4. Πανεπιστήμιο/Πτυχίο □
5. Μεταπτυχιακό □
6. Διδακτορικό □

**A6. Ποιο είναι το θρήσκευμα σας;**

1. Χριστιανή Ορθόδοξη □
2. Χριστιανή Καθολική □
3. Μάρτυρας του Ιεχωβά □
4. Άλλο □ (Προσδιορίστε): ……………………..

**A7. Ποια είναι η οικογενειακή σας κατάσταση;**

1. Άγαμη □
2. Σε συμβίωση / σε σχέση □
3. Παντρεμένη □
4. Σε διάσταση □
5. Διαζευγμένη □
6. Χήρα □

**Α8. Παρακαλώ σημειώστε την επαγγελματική σας κατάσταση.**

1. Ιδιωτική υπάλληλος □
2. Δημόσιος υπάλληλος □
3. Ελεύθερη επαγγελματίας □
4. Άνεργη □
5. Οικιακά □
6. Συνταξιούχος □
7. Άλλο: □ (Προσδιορίστε): ……………………..

**Α9. Το μηνιαίο σας ακάθαρτο εισόδημα είναι (ευρώ):**

1. Δεν έχω εισόδημα □
2. Λιγότερο από 500 □
3. 501-1000 □
4. 1001-1500 □
5. 1501-2000 □
6. Περισσότερο από 2001 □

**Β. ΙΣΤΟΡΙΚΟ ΕΓΚΥΜΟΣΥΝΗΣ**

**B1. Είστε έγκυος αυτή την χρονική περίοδο;**

1. Ναι □
2. Όχι □
3. Δεν είμαι σίγουρη □

**Β2. Είχατε τουλάχιστον μία εγκυμοσύνη που διήρκησε περισσότερο από 6 μήνες;**

1. Ναι □
2. Όχι □

**Β3. Πόσα παιδιά έχετε;**

1. 0 □
2. 1 □
3. 2 □
4. 3 □
5. 4 ή περισσότερα □

**Β4. Είχατε στο παρελθόν (πριν από την εγκυμοσύνη) υψηλή αρτηριακή πίεση;**

1. Ναι □
2. Όχι □
3. Δεν είμαι σίγουρη □

**Β5. Έχετε διαγνωστεί (πριν από την εγκυμοσύνη) από γιατρό με υπέρταση;**

1. Ναι □
2. Όχι □
3. Δεν είμαι σίγουρη □

**Β6. Κατά τη διάρκεια οποιασδήποτε εγκυμοσύνης (που διήρκησε περισσότερο από 6 μήνες), σας ανάφερε ποτέ ο ιατρός ότι είχατε υψηλή αρτηριακή πίεση ή υπέρταση;**

1. Ναι, μόνο στην πρώτη εγκυμοσύνη □
2. Όχι, στην πρώτη εγκυμοσύνη, αλλά σε επόμενη □
3. Στην πρώτη εγκυμοσύνη και τουλάχιστον σε μία επόμενη □
4. Όχι, σε καμία εγκυμοσύνη □

**Β7. Κατά τη διάρκεια οποιασδήποτε εγκυμοσύνης στην οποία εμφανίσατε αυξημένη αρτηριακή πίεση, είχατε:**

1. Υψηλά επίπεδα πρωτεΐνης στα ούρα □
2. Επιληπτικές κρίσεις ή σπασμούς □
3. Προεκλαμψία, εκλαμψία ή τοξαιμία της εγκυμοσύνης □

**Β8. Κατά τη διάρκεια οποιασδήποτε εγκυμοσύνης παρουσιάσατε προεκλαμψία, εκλαμψία, ή τοξαιμία;**

1. Ναι □
2. Όχι □

**Β9. Πόσο χρονών ήσασταν όταν εμφανίσατε για πρώτη φορά υψηλή αρτηριακή πίεση που σχετιζόταν με την εγκυμοσύνη;**

1. Ηλικία ……………..

**Γ. ΕΡΩΤΗΣΕΙΣ ΠΟΥ ΑΦΟΡΟΥΝ ΤΗ ΓΕΝΙΚΟΤΕΡΗ ΥΓΕΙΑ**

**Γ1. Είχατε ποτέ κάποιο από τα ακόλουθα κατά τη διάρκεια, πριν ή μετά την εγκυμοσύνη;**

1. Υψηλή αρτηριακή πίεση □
2. Διαβήτη □
3. Υψηλός Δείκτης Μάζας Σώματος (Παχυσαρκία) □
4. Εγκεφαλικό επεισόδιο □
5. Καρδιαγγειακό επεισόδιο □
6. Νεφρικά προβλήματα □
7. Κανένα από τα πιο πάνω / κανένα σημαντικό πρόβλημα υγείας □
8. Άλλο □ (Προσδιορίστε): …………………………..

**Γ2. Από τη λίστα πιο κάτω, τι ισχύει σήμερα για εσάς (επιλέξτε όλα σας αφορούν);**

1. Κάπνισμα □
2. Παχυσαρκία □
3. Υψηλή κατανάλωση αλκοόλ □
4. Καθιστική ζωή / Έλλειψη φυσικής δραστηριότητας □
5. Αγχωτική εργασία □
6. Σπάνια ή καθόλου κατανάλωση φρέσκων φρούτων και λαχανικών □
7. Οικογενειακό ιστορικό καρδιαγγειακών νόσων □
8. Υψηλή χοληστερόλη □
9. Υψηλή αρτηριακή πίεση □
10. Διαβήτης □
11. Κανένα από τα πιο πάνω □
12. Άλλο □ (Προσδιορίστε): ……………….……………

**Γ3. Σε ποιο βαθμό ανησυχείτε ότι μπορεί να διατρέχετε κίνδυνο για τα πιο κάτω νοσήματα;**

|  | Καθόλου | Λίγο | Αρκετά | Πολύ | Πάρα πολύ |
| --- | --- | --- | --- | --- | --- |
| Καρδιαγγειακές παθήσεις |  |  |  |  |  |
| Προβλήματα βάρους / Παχυσαρκία |  |  |  |  |  |
| Καρκίνος (εκτός από καρκίνο μαστού) |  |  |  |  |  |
| Καρκίνος του μαστού |  |  |  |  |  |
| Υπέρταση / Υψηλή αρτηριακή πίεση |  |  |  |  |  |
| Οστεοπόρωση |  |  |  |  |  |
| Διαβήτης |  |  |  |  |  |
| Άνοια και Νόσος Alzheimer |  |  |  |  |  |

**Γ4. Πόσο συχνά μιλάτε με το ιατρό σας για καθένα από τα πιο κάτω;**

|  | Δεν επισκέπτομαι ιατρό | Ποτέ | Σπάνια | Μερικές φορές | Συχνά | Πολύ συχνά |
| --- | --- | --- | --- | --- | --- | --- |
| Καρδιαγγειακές παθήσεις |  |  |  |  |  |  |
| Προβλήματα βάρους / Παχυσαρκία |  |  |  |  |  |  |
| Καρκίνος (εκτός από καρκίνο μαστού) |  |  |  |  |  |  |
| Καρκίνος του μαστού |  |  |  |  |  |  |
| Υπέρταση / Υψηλή αρτηριακή πίεση |  |  |  |  |  |  |
| Οστεοπόρωση |  |  |  |  |  |  |
| Διαβήτης |  |  |  |  |  |  |
| Άνοια και Νόσος Alzheimer |  |  |  |  |  |  |

**Γ5. Πόσο συχνά ελέγχετε την αρτηριακή σας πίεση;**

1. Ποτέ □
2. Μια φορά το χρόνο □
3. 2-3 φορές τον χρόνο □
4. Μια φορά το μήνα □
5. Μια φορά την εβδομάδα □
6. Περισσότερο από μια φορά την εβδομάδα □

**Γ6. Πόσο συχνά ελέγχετε τα επίπεδα γλυκόζης σας;**

1. Ποτέ □
2. Μια φορά το χρόνο □
3. 2-3 φορές τον χρόνο □
4. Μια φορά το μήνα □
5. Μια φορά την εβδομάδα □
6. Περισσότερο από μια φορά την εβδομάδα □

**Δ. ΕΡΩΤΗΣΕΙΣ ΣΧΕΤΙΚΑ ΜΕ ΤΙΣ ΓΝΩΣΕΙΣ ΚΑΙ ΑΝΤΙΛΗΨΕΙΣ ΓΙΑ ΤΙΣ ΥΠΕΡΤΑΣΙΚΕΣ ΔΙΑΤΑΡΑΧΕΣ ΚΥΗΣΗΣ**

**Δ1. Κατά τη γνώμη σας, ποιο από τα παρακάτω εάν υπάρχει, θα μπορούσε να αυξήσει την πιθανότητα σε μια γυναίκα να πάθει καρδιαγγειακή νόσο; Επιλέξτε όλα όσα ισχύουν.**

| Να είναι υπέρβαρη/παχύσαρκη |  |
| --- | --- |
| Να καταναλώνει υψηλές ποσότητες αλκοόλ |  |
| Να έχει ανθυγιεινή διατροφή |  |
| Να έχει οικογενειακό ιστορικό καρδιαγγειακών παθήσεων |  |
| Να έχει υψηλή αρτηριακή πίεση |  |
| Να καπνίζει |  |
| Να έχει υψηλή χοληστερόλη |  |
| Να έχει ελάχιστη ή καθόλου φυσική δραστηριότητα |  |
| Να έχει διαβήτη |  |
| Να είχε επιπλοκές κατά την εγκυμοσύνη σχετιζόμενες με υπερτασικές διαταραχές, όπως προεκλαμψία |  |
| Να έχει αυτοάνοσες ασθένειες, όπως λύκος ή ρευματοειδής αρθρίτιδα |  |
| Να έχει / είχε πρώιμη εμμηνόπαυση |  |
| Να έχει / είχε ακανόνιστη έμμηνο ρήση |  |
| Δεν είμαι σίγουρη |  |

**Δ2. Κατά τη γνώμη σας οι καρδιαγγειακές ασθένειες οφείλονται:**

| Σε γενετική προδιάθεση (οικογενειακό ιστορικό) |  |
| --- | --- |
| Στον τρόπο ζωής (π.χ. άσκηση, διατροφή) |  |
| Και τα δύο πιο πάνω |  |

**Δ3. Το υπερβολικό βάρος είναι ένας σημαντικός παράγοντας κινδύνου για την ανάπτυξη καρδιαγγειακών παθήσεων;**

| Διαφωνώ απόλυτα |  |
| --- | --- |
| Διαφωνώ |  |
| Ούτε συμφωνώ ούτε διαφωνώ |  |
| Συμφωνώ |  |
| Συμφωνώ απόλυτα |  |

**Ε. ΕΡΩΤΗΣΕΙΣ ΠΟΥ ΑΦΟΡΟΥΝ ΤΗΝ ΕΝΗΜΕΡΩΣΗ ΣΧΕΤΙΚΑ ΜΕ ΤΙΣ ΥΠΕΡΤΑΣΙΚΕΣ ΔΙΑΤΑΡΑΧΕΣ ΣΤΗΝ ΚΥΗΣΗ**

**Ε1. Πριν από τη διάγνωση σας γνωρίζατε για τις υπερτασικές διαταραχές (π.χ. προεκλαμψία) που μπορεί να παρουσιαστούν κατά την εγκυμοσύνη;**

1. Ναι □
2. Όχι □

**Ε2. Με ποιο τρόπο είχατε ενημερωθεί πριν από τη διάγνωση σας;**

1. Προσωπικός ιατρός □
2. Γυναικολόγος / Μαιευτήρας □
3. Διαδίκτυο □
4. Κοινωνικό περιβάλλον □
5. Άλλο □ (Προσδιορίστε): …………………………
6. Κανένα τρόπο □

**Ε3. Πότε σας έχει μιλήσει κάποιος πάροχος υγειονομικής περίθαλψης για τους μελλοντικούς κινδύνους στην υγεία σας μετά από τη διάγνωση σας;**

1. Πριν τον τοκετό □
2. Αμέσως μετά τον τοκετό □
3. Εντός 6 εβδομάδων μετά τον τοκετό □
4. Από 6 εβδομάδες μέχρι 6 μήνες μετά τον τοκετό □
5. Από 6 μήνες μέχρι 1 χρόνο μετά τον τοκετό □
6. Σε περισσότερο από 1 χρόνο □
7. Δεν θυμάμαι □

**Ε4. Κατά τη διάρκεια της εγκυμοσύνης σας λαμβάνεται κάποια προληπτική θεραπεία (π.χ. ήπια άσκηση ή αποφυγή καπνίσματος και αλκοόλ);**

1. Φαρμακευτική αγωγή □
2. Βελτιωμένη διατροφή □
3. Καμία προληπτική θεραπεία □

**Ε5. Έχετε ενημερωθεί ότι μετά από εγκυμοσύνη με υπερτασικές διαταραχές (π.χ. προεκλαμψία) μπορούν να υπάρξουν μελλοντικοί κίνδυνοι στην υγείας σας;**

1. Ναι □
2. Όχι □
3. Δεν είμαι σίγουρη □

**Ε6.** **Με ποιο τρόπο ενημερωθήκατε για τυχόν μελλοντικούς κινδύνους;**

1. Προσωπικός ιατρός □
2. Γυναικολόγος / Μαιευτήρας □
3. Μαία □
4. Διαδίκτυο □
5. Κοινωνικό περιβάλλον □
6. Άλλο □ (Προσδιορίστε): …………………………
7. Κανένα τρόπο □

**Ε7. Ποιοι είναι οι μελλοντικοί κίνδυνοι που γνωρίζετε;**

1. Υπέρταση □
2. Καρδιαγγειακές νόσοι □
3. Νεφρική νόσος □
4. Διαβήτης □
5. Άλλο □ (Προσδιορίστε): …………………………
6. Δεν γνωρίζω κανένα □

**Ε8. Έχετε λάβει κάποιες από τις πιο κάτω συμβουλές προ συμπτωματικού ελέγχου μετά από την τελευταία σας εγκυμοσύνη στην οποία αντιμετωπίσατε προβλήματα υπερτασικών διαταραχών (π.χ. προεκλαμψία);**

1. Έλεγχος αρτηριακής υπέρτασης □
2. Έλεγχος χοληστερόλης □
3. Έλεγχος γλυκόζης □
4. Παρότρυνση για διακοπή του καπνίσματος □
5. Παρότρυνση για έλεγχο του σωματικού σας βάρους □
6. Συμβουλή για συχνή σωματική άσκηση □
7. Συμβολή για υγιεινή διατροφή □

**Ε9. Ακολουθείτε οποιαδήποτε προληπτική ενέργεια που να σχετίζεται με τους μελλοντικούς κινδύνους;**

1. Παρακολούθηση από ειδικό ιατρό □
2. Βελτιωμένη διατροφή □
3. Άσκηση
4. Αποφυγή καπνίσματος
5. Αποφυγή υψηλής κατανάλωσης αλκοόλ
6. Φαρμακευτική αγωγή □
7. Καμία προληπτική ενέργεια □
8. Άλλο □ (Προσδιορίστε): …………………………

**Ε10. Δεδομένου ότι στην εγκυμοσύνη σας αντιμετωπίσατε προβλήματα υπερτασικών διαταραχών** **(π.χ. προεκλαμψία), μετά τον τοκετό απευθυνθήκατε σε οποιοδήποτε από τους πιο κάτω; Επιλέξτε όλα όσα ισχύουν.**

1. Καρδιολόγος □
2. Νεφρολόγος □
3. Γενικός ιατρός / Προσωπικός ιατρός □
4. Γυμναστήριο □
5. Διατροφολόγος □
6. Δεν θυμάμαι □
7. Άλλο □ (Προσδιορίστε): ……………………………..

**Ε11. Πότε αρχίσατε να λαμβάνετε τα μέτρα πρόληψης;**

1. Αμέσως μετά τον τοκετό □
2. 3 έως 12 μήνες μετά τον τοκετό □
3. 1 έως 3 χρόνια μετά τον τοκετό □
4. 4-6 χρόνια μετά τον τοκετό □
5. Ποτέ □

**Ε12. Πόσο συχνά ρωτάτε τον ιατρό σας για την υγεία της καρδιάς σας;**

1. Δεν επισκέπτομαι ιατρό □
2. Ποτέ □
3. Σπάνια □
4. Μερικές φορές □
5. Συχνά □
6. Πολύ συχνά □

**Ε13. Τους τελευταίους 12 μήνες έχετε ελέγξει την καρδία σας σε κάποιο ιατρό;**

1. Ναι □
2. Όχι □
3. Δεν είμαι σίγουρη □

**Ε14. Εάν έχετε ελέγξει την καρδία σας τους τελευταίους 12 μήνες, συζητήσατε τα αποτελέσματα της εξέτασης με τον ιατρό σας;**

1. Ναι □
2. Όχι □
3. Δεν είμαι σίγουρη □

**ΤΕΛΟΣ ΕΡΩΤΗΜΑΤΟΛΟΓΙΟΥ**

**Σας ευχαριστούμε πολύ για το χρόνο που διαθέσατε και τη σημαντική βοήθειά σας για την υλοποίηση της παρούσας μελέτης.**
