# Supplementary material for: Assessing awareness of long-term health risks among women with a history of preeclampsia: a cross-sectional study
Source: Front Med (Lausanne). 2023 Nov 7;10:1236314. doi: 10.3389/fmed.2023.1236314 (PMC10662303; doi:10.3389/fmed.2023.1236314)
Supplement: Supplementary file 3 [file Data_Sheet_2.docx]

| **Supplementary file 2:** Sensitivity Analysis - Time Since Study Conduction | | | |
| --- | --- | --- | --- |
| **Characteristics** | **Preeclampsia group** | | |
|  | **Time since study conduction** | | |
|  | **≤ 3 years**  (N=73) | **> 3 years**  (N=66) | **p-value** |
| **Sociodemographic characteristics** | | | |
| **Age**, Median (IQR) | 33 (30, 37) | 38.5 (36, 42) | **<0.001^o^** |
| **Country**, N^a^ (%) | | | |
| Cyprus | 21 (37.5) | 35 (62.5) | **0.009^p^** |
| Greece | 51 (63.0) | 30 (37.0) |  |
| Other | 1 (100.0) | 0 (0.0) |  |
| **Geographical area**, N^b^ (%) | | | |
| Nicosia | 9 (33.3) | 18 (66.7) | **0.011^p^** |
| Athens | 24 (72.7) | 9 (27.3) |  |
| Limassol | 9 (40.9) | 13 (59.1) |  |
| Other in Greece | 15 (68.2) | 7 (31.8) |  |
| Larnaca | 3 (37.5) | 5 (62.5) |  |
| Thessaloniki | 5 (71.4) | 2 (28.6) |  |
| Abroad | 0 (0.0) | 3 (100.0) |  |
| Paphos | 0 (0.0) | 1 (100.0) |  |
| Ammochostos | 1 (100.0) | 0 (0.0) |  |
| **Residency**, N^c^ (%) | | | |
| Urban | 60 (54.5) | 50 (45.5) | 0.351^p^ |
| Rural | 13 (44.8) | 16 (55.2) |  |
| **Educational level**, N^c^ (%) | | | |
| Secondary education | 12 (57.1) | 9 (42.9) | 0.123^p^ |
| Undergraduate education | 42 (59.2) | 29 (40.8) |  |
| Postgraduate education | 19 (40.4) | 28 (59.6) |  |
| **Religion**, N^d^ (%) | | | |
| Christian Orthodox | 72 (54.5) | 60 (45.5) | 0.319^p^ |
| No religion/Other | 1 (20.0) | 4 (80.0) |  |
| **Marital status**, N^c^ (%) | | | |
| Married/In cohabitation | 69 (53.5) | 60 (46.5) | 0.117^p^ |
| Unmarried | 2 (100.0) | 0 (0.0) |  |
| Divorced/separated/widowed | 2 (25.0) | 6 (75.0) |  |
| **Occupation**, N^c^ (%) | | | |
| Private employee | 35 (53.0) | 31 (47.0) | **0.003^p^** |
| State employee | 10 (31.2) | 22 (68.8) |  |
| Freelancer | 5 (41.7) | 7 (58.3) |  |
| Unemployed | 13 (86.7) | 2 (13.3) |  |
| Housewife | 10 (76.9) | 3 (23.1) |  |
| Retired | 0 (0.0) | 1 (100.0) |  |
| **Monthly income**, N^e^ (%) | | | |
| No income | 18 (78.3) | 5 (21.7) | **0.002^p^** |
| Less than €500 | 2 (40.0) | 3 (60.0) |  |
| €501- €1000 | 30 (63.8) | 17 (36.2) |  |
| €1001- €1501 | 7 (31.8) | 15 (68.2) |  |
| €1501- €2000 | 10 (47.6) | 11 (52.4) |  |
| More than €2001 | 4 (22.2) | 14 (77.8) |  |
| **Information** **on concerns about future disease risks.** | | | |
| **To what extent are you concerned that you may be at risk for the following diseases?** | | | |
| **Cardiovascular diseases**, N^f^ (%) | | | |
| Not at all | 28 (68.3) | 13 (31.7) | 0.128^p^ |
| Little | 25 (49.0) | 26 (51.0) |  |
| Enough | 10 (37.0) | 17 (63.0) |  |
| Very | 6 (60.0) | 4 (40.0) |  |
| Extremely | 2 (50.0) | 2 (50.0) |  |
| **Weight problems/Obesity**, N^f^ (%) | | | |
| Not at all | 37 (68.5) | 17 (31.5) | 0.060^p^ |
| Little | 15 (46.9) | 17 (53.1) |  |
| Enough | 14 (40.0) | 21 (60.0) |  |
| Very | 2 (33.3) | 4 (66.7) |  |
| Extremely | 3 (50.0) | 3 (50.0) |  |
| **Cancer (except breast cancer)**, N^g^ (%) | | | |
| Not at all | 27 (61.4) | 17 (38.6) | 0.405^p^ |
| Little | 23 (47.9) | 25 (52.1) |  |
| Enough | 14 (46.7) | 16 (53.3) |  |
| Very | 2 (100.0) | 0 (0.0) |  |
| Extremely | 2 (50.0) | 2 (50.0) |  |
| **Breast cancer**, N^h^ (%) | | | |
| Not at all | 25 (55.6) | 20 (44.4) | 0.738^p^ |
| Little | 22 (53.7) | 19 (46.3) |  |
| Enough | 16 (51.6) | 15 (48.4) |  |
| Very | 4 (80.0) | 1 (20.0) |  |
| Extremely | 1 (33.3) | 2 (66.7) |  |
| **Hypertension/High blood pressure**, N^i^ (%) | | | |
| Not at all | 16 (53.3) | 14 (46.7) | 0.690^p^ |
| Little | 24 (57.1) | 18 (42.9) |  |
| Enough | 16 (44.4) | 20 (55.6) |  |
| Very | 9 (64.3) | 5 (35.7) |  |
| Extremely | 6 (60.0) | 4 (40.0) |  |
| **Osteoporosis**, N^j^ (%) | | | |
| Not at all | 40 (66.7) | 20 (33.3) | 0.071^p^ |
| Little | 17 (39.5) | 26 (60.5) |  |
| Enough | 8 (44.4) | 10 (55.6) |  |
| Very | 1 (33.3) | 2 (66.7) |  |
| Extremely | 1 (50.0) | 1 (50.0) |  |
| **Diabetes**, N^b^ (%) | | | |
| Not at all | 31 (57.4) | 23 (42.6) | 0.679^p^ |
| Little | 14 (46.7) | 16 (53.3) |  |
| Enough | 15 (57.7) | 11 (42.3) |  |
| Very | 3 (37.5) | 5 (62.5) |  |
| Extremely | 4 (66.7) | 2 (33.3) |  |
| **Dementia/Alzheimer**, N^k^ (%) | | | |
| Not at all | 36 (60.0) | 24 (40.0) | 0.146^p^ |
| Little | 22 (56.4) | 17 (43.6) |  |
| Enough | 7 (33.3) | 14 (66.7) |  |
| Very | 1 (50.0) | 1 (50.0) |  |
| Extremely | 1 (20.0) | 4 (80.0) |  |
| **Participant-doctor communication and health checks.** | | | |
| **How often do you talk to your doctor about any of the following?** | | | |
| **Cardiovascular diseases**, N^l^ (%) | | | |
| Not at all | 46 (56.8) | 35 (43.2) | 0.330^p^ |
| Little | 13 (40.6) | 19 (59.4) |  |
| Enough | 7 (43.7) | 9 (56.3) |  |
| Very | 3 (75.0) | 1 (25.0) |  |
| Extremely | 1 (100.0) | 0 (0.0) |  |
| **Weight problems/Obesity**, N^m^ (%) | | | |
| Not at all | 46 (60.5) | 30 (39.5) | 0.055^p^ |
| Little | 11 (33.3) | 22 (66.7) |  |
| Enough | 9 (52.9) | 8 (47.1) |  |
| Very | 2 (66.7) | 1 (33.3) |  |
| Extremely | 0 (0.0) | 2 (100.0) |  |
| **Cancer (except breast cancer)**, N^n^ (%) | | | |
| Not at all | 52 (55.3) | 42 (44.7) | 0.520^p^ |
| Little | 12 (42.9) | 16 (57.1) |  |
| Enough | 3 (50.0) | 3 (50.0) |  |
| Very | 1 (100.0) | 0 (0.0) |  |
| Extremely | 0 (0.0) | 0 (0.0) |  |
| **Breast cancer**, N^g^ (%) | | | |
| Not at all | 47 (56.0) | 37 (44.0) | 0.588^p^ |
| Little | 16 (47.1) | 18 (52.9) |  |
| Enough | 4 (44.4) | 5 (55.6) |  |
| Very | 1 (100.0) | 0 (0.0) |  |
| Extremely | 0 (0.0) | 0 (0.0) |  |
| **Hypertension/High blood pressure**, N^f^ (%) | | | |
| Not at all | 28 (53.8) | 24 (46.2) | 0.717^p^ |
| Little | 22 (51.2) | 21 (48.8) |  |
| Enough | 16 (53.3) | 14 (46.7) |  |
| Very | 1 (25.0) | 3 (75.0) |  |
| Extremely | 3 (75.0) | 1 (25.0) |  |
| **Osteoporosis**, N^h^ (%) | | | |
| Not at all | 58 (56.3) | 45 (43.7) | 0.134^p^ |
| Little | 6 (33.3) | 12 (66.7) |  |
| Enough | 3 (75.0) | 1 (25.0) |  |
| Very | 0 (0.0) | 0 (0.0) |  |
| Extremely | 0 (0.0) | 0 (0.0) |  |
| **Diabetes**, N^g^ (%) | | | |
| Not at all | 48 (52.8) | 43 (47.2) | 0.150^p^ |
| Little | 4 (26.7) | 11 (73.3) |  |
| Enough | 10 (66.7) | 5 (33.3) |  |
| Very | 1 (50.0) | 1 (50.0) |  |
| Extremely | 4 (80.0) | 1 (20.0) |  |
| **Dementia/Alzheimer**, N^k^ (%) | | | |
| Not at all | 62 (54.9) | 51 (45.1) | 0.520^p^ |
| Little | 4 (36.4) | 7 (63.6) |  |
| Enough | 1 (50.0) | 1 (50.0) |  |
| Very | 1 (100.0) | 0 (0.0) |  |
| Extremely | 0 (0.0) | 0 (0.0) |  |
| ^a^N=138; ^b^N=124; ^c^N=139; ^d^N=137; ^e^N=136; ^f^N=133; ^g^N=128; ^h^N=125; ^i^N=132; ^j^N=126; ^k^N=127; ^l^N=134; ^m^N=131; ^n^N=129; ^o^Differences between groups were tested using Kolmogorov-Smirnov test; ^p^Differences between groups were tested using chi^2^ test. Bold values indicate statistically significant associations. | | | |
